# The Accuracy of Pulse Oxygen Saturation, Heart Rate, Blood Pressure, and Respiratory Rate Raised by a Contactless Telehealth Portal: Validation Study

**DOI:** 10.2196/55361

**Published:** 2024-06-28

**Authors:** Julian Gerald Dcruz, Paichang Yeh

**Affiliations:** 1 Docsun Biomedical Holdings, Inc Saint Petersburg, FL United States

**Keywords:** medical devices, mHealth, vital signs, measurements validity, validation, validity, device, devices, vital, vitals, accuracy, pulse, oxygen, saturation, heart rate, blood pressure, respiration, respiratory, telehealth, telemedicine, eHealth, e-health, self-check, self-checker, breathing, portal, portals, self-checking, self-monitor, self-monitoring

## Abstract

**Background:**

The traditional measurement of heart rate (HR), oxygen saturation (SpO_2_), blood pressure (BP), and respiratory rate (RR) via physical examination can be challenging, and the recent pandemic has accelerated trends toward telehealth and remote monitoring. Instead of going to the physician to check these vital signs, measuring them at home would be more convenient. Vital sign monitors, also known as physiological parameter monitors, are electronic devices that measure and display biological information about patients under constant monitoring.

**Objective:**

The purpose of this study was to validate the accuracy of the pulse SpO_2_, HR, BP, and RR raised by Docsun Telehealth Portal by comparing it with approved medical devices.

**Methods:**

This is a noninvasive, self-check, system-based study conducted to validate the detection of vital signs (SpO_2_, HR, BP, and RR) raised by Docsun Telehealth Portal. The input for software processing involves facial screening without any accessories on the face, scanning directly through the software application portal. The participant’s facial features are detected and screened for the extraction of necessary readings.

**Results:**

For the validation of HR, SpO_2_, BP, and RR measurements, the main outcomes were the mean of the absolute difference between the respective investigational devices and the reference values as well as the absolute percentage difference between the respective investigational devices and the reference values. If the HR was within ±10% of the reference standard or 5 beats per minute, it was considered acceptable for clinical purposes. The average absolute difference between the Docsun Telehealth Portal and the reference values was 1.41 (SD 1.14) beats per minute. The mean absolute percentage difference was 1.69% (SD 1.37). Therefore, the Docsun Telehealth Portal met the predefined accuracy cutoff for HR measurements. If the RR was within ±10% of the reference standard or 3 breaths per minute, it was considered acceptable for clinical purposes. The average absolute difference between the Docsun Telehealth Portal and the reference values was 0.86 breaths per minute. The mean absolute percentage difference was 4.72%. Therefore, the Docsun Telehealth Portal met the predefined accuracy cutoff for RR measurements. SpO_2_ levels were considered acceptable if the average absolute difference between the Docsun Telehealth Portal and the reference values was ±3%. The mean absolute percentage difference was 0.59%. Therefore, the Docsun Telehealth Portal met the predefined accuracy cutoff for SpO_2_ measurements. The Docsun Telehealth Portal predicted systolic BP with an accuracy of 94.81% and diastolic BP with an accuracy of 95.71%.

**Conclusions:**

The results of the study show that the accuracy of the HR, BP, SpO_2_, and RR values raised by the Docsun Telehealth Portal, compared against the clinically approved medical devices, proved to be accurate by meeting predefined accuracy guidelines.

## Introduction

### Background

Heart rate (HR), blood pressure (BP), oxygen saturation (SpO_2_), and respiratory rate (RR) are the 4 cardinal vital signs. The measurement of these vital signs is the starting point of physical assessment for both health and wellness. The traditional measurement of these vital signs through physical examination can be challenging, and the recent pandemic has accelerated trends toward telehealth and remote monitoring. Instead of going to the physician to check HR, SpO_2_, BP, body temperature, and RR, it would be convenient to measure them at home [1]. Vital sign monitors, also known as physiological parameter monitors, are electronic devices that measure and display biological information about patients under constant monitoring [2]. The purpose of this study was to validate the HR, BP, SpO_2_, and RR accuracy of the Docsun Telehealth Portal [3], compared to existing approved medical devices.

HR, RR, and SpO_2_ are 3 of the 5 vital signs indicating the criticality of a patient’s condition (aside from BP and body temperature). If any of these vital signs deteriorate, it indicates that the patient requires immediate medical intervention [4]. With the introduction of photoplethysmography-based pulse oximeters, HR and SpO_2_ can easily be monitored using a single device, and respiration is often monitored visually or through separate devices as required. A single device that can monitor all 3 signs is a desirable diagnostic tool for emergency medicine and for the monitoring as well as diagnosis of other diseases [5].

### Objectives

The aim of this study was to validate the accuracy of the SpO_2_, HR, BP, and RR raised by Docsun Telehealth compared against clinically approved medical devices.

## Methods

### Ethical Considerations

#### Human Subject Research Ethics Review and Approvals

This study underwent ethical review and approval by the appropriate authorities at the clinic (Oloolua Dispensary, Kenya; DCM-HM-02) and followed the principles outlined in the Declaration of Helsinki and Good Clinical Practice guidelines. The study protocol, including all amendments, and the patient information sheets were reviewed and approved by authorized clinical investigators. No exemptions were granted for this study, and all procedures adhered to ethical standards for human subject research.

The study was conducted in accordance with the Code of Federal Regulations (CFR) for nonsignificant risk medical device studies, following the Food and Drug Administration’s Good Clinical Practice regulations at 21 CFR parts 50, 56, and 812. It also adhered to the relevant International Organization for Standardization (ISO) 14155, and the International Conference on Harmonization (ICH) of Technical Requirements for Registration of Pharmaceuticals for Human Use “Good Clinical Practice: Consolidated Guidance” (ICH E6) [6].

#### Informed Consent

All participants were enrolled between March 7 and March 12, 2022. Prior to participation, all participants provided written informed consent after a detailed explanation of the study procedures and associated risks. The informed consent process ensured that participants understood their voluntary participation, the purpose of the study, potential risks and benefits, and the confidentiality of their data. For studies identified as human subject research, informed consent was obtained from all participants based on ethical guidelines. Any waivers of informed consent did not apply to this study, as all participants provided explicit consent for their involvement.

#### Privacy and Confidentiality Protection

Measures were implemented to safeguard the privacy and confidentiality of participants’ data throughout the study. Study data were anonymized or deidentified to ensure that individual participants could not be identified from the collected data. Additionally, access to identifiable information was restricted to authorized personnel only, and all electronic data were securely stored on password-protected servers. Any physical records containing participant information were stored in locked filing cabinets accessible only to designated study personnel. Participants’ confidentiality was upheld by using unique identifiers instead of personal identifiers in study documentation. Moreover, any publication or presentation of study results ensured that no identifiable information about participants was disclosed, maintaining their anonymity and confidentiality.

#### Compensation

No compensation was provided to participants for their involvement in this study. Participation was voluntary, and participants did not receive any financial incentives or compensation of any kind for their participation. This decision was made to minimize any potential influence on participants’ motivations for involvement and to uphold the integrity of the study’s findings.

### Device Description

The Docsun Telehealth Portal uses a custom algorithm and optical coherence tomography technology to capture micrometer-resolution, 2D, and 3D images from within biological tissue. The noninvasive and highly repeatable optical coherence tomography procedure provides a fully automated screening process, which helps monitor health signs. The software panel allows the user to upload a prerecorded video or use the webcam on the device to run the detection. The software algorithm runs the video recording and produces the detection results within 60 seconds showing the user’s pulse and RR.

The artificial intelligence (AI) system checks if the features are visible and under ambient lighting conditions, and if not, a message will appear to ask the user to position their face at a more visible angle. The detection runs for 60 seconds, after which the outcome will be visible with readings of pulse rate and RR. The user can use the health report for self-analysis or share it for further medical consultation. The users have access to their previous readings on our software, which they can use as a reference for self-analysis.

### Indications for Use

The Docsun Telehealth Portal is a web application intended to be used for monitoring, displaying, reviewing, and storing multiple physiological patient parameters, such as SpO_2_, HR, BP, and RR. The Docsun Telehealth Portal is intended for use in a professional setting or at home and is a noninvasive vital sign monitoring system. The data and results provided by this device are for precheck screening purposes only and cannot be directly used for diagnosis or treatment.

The device is indicated for use on humans 18 years of age or older who do not require critical care or continuous vital sign monitoring.

The device is not intended to be the sole method of checking the physical health of a person.

### Reference Equipment

A pulse oximeter and digital BP monitor were used as a reference standard device for obtaining the functional values (for HR, BP, SpO_2_, and RR) during the study.

### Validation Equipment

This noninvasive, self-check, system-based study was conducted to validate the detection of vital signs (ie, HR, BP, SpO_2_, and RR) using an AI tool. Data for this study were collected at a single collaborating site approved by Docsun. The data of patients visiting the site were collected based on the study criteria after obtaining their informed consent.

This study aims to collect basic vital signs information on the current health status to generate a diagnostic report using an AI tool. The input for the software processing involves a facial screening without any accessories on the face, directly scanned through the software application portal. The participant’s facial features are detected and screened to extract necessary readings.

The study setup was designed so that the participant was always at a distance of 2 meters from the study staff. The brochures and participant signature logs were placed near the study screening space. Basic vital signs, such as HR and RR, were measured using a pulse oximeter and digital blood pressure monitor as reference devices. The readings were noted in a case report form. A webcam was installed at a convenient distance from the participant for facial screening. Then the participant was asked to look into the webcam for software screening. The participant was asked not to make any sudden movements but to breathe normally for 60 seconds.

The software provided a health report, and the report’s upload time was noted for study data collection purposes. The study area was then sanitized using a spray and fumigation machine before the entry of the next participant.

### Inclusion Criteria

Participants aged 18 years and older, with cardiovascular and respiratory diseases, and able to provide informed consent were included in the study.

### Exclusion Criteria

Participants wearing contact lenses and those who did not require critical medical care were excluded from the study.

### Subject Demographics

Participant demographics are shown in Table 1.

**Table 1:** Participant demographics (N=100).

Characteristics		Values, n (%)
**Sex**		
	Female	42 (42)
	Male	58 (58)
	Total	100 (100)
**Age (years)**		
	18-20	11 (11)
	20-40	45 (45)
	41-65	44 (44)

### Data Analysis

Microsoft Excel was used as the data analysis tool. Measurements were paired observations, as follows: the algorithm-estimated HR, BP, SpO_2_, and RR as well as the reference HR, BP, SpO_2_, and RR from the reference devices. The absolute error of each paired measurement was calculated as the absolute value of the difference between the algorithm-estimated and the reference HR, SpO_2_, BP, and RR values.

The mean absolute error was the mean value of all absolute errors. Similarly, the absolute error from each paired measurement was divided by the reference value for that measurement and multiplied by 100 to produce the absolute percentage error. The mean absolute percentage error was the mean value for all absolute percentage error values. The values from each participant were treated as statistically independent, as the clustering effects were observed to be minimal.

For the HR, BP, SpO_2_, and RR measurement validation, the main outcome was the mean of the absolute difference between the respective investigational devices and the reference values as well as the absolute percentage difference between the respective investigational devices and the reference values.

HR measurements were considered accurate if the mean absolute difference was within either ±10 % or ±5 beats per minute (bpm), depending on which of the two was greater.

RR measurements were considered accurate if the mean absolute difference was within either ±10 % or ±3 bpm.

SpO_2_ measurements were considered accurate if the mean absolute difference was ≤3.0%

For BP measurements, a tolerable error of ≤10 mm Hg and an estimated probability of that error of at least 85% was acceptable as a compromise, considering the performance of currently available BP monitors. This was compatible with the current American National Standards Institute, Association for the Advancement of Medical Instrumentation, the ISO requirements, and the requirements of the revised European Society of Hypertension International Protocol, allowing for a 10 mm Hg error with a frequency of 12% to 18%.

The main outcome data were visualized using Bland-Altman plots. The Bland-Altman analysis is a simple and efficient method to assess the agreement between two measurements in clinical studies [7]. In addition, correlation analyses and scatter plots were used to assess the relation between the respective investigational devices and the reference values.

## Results

### Principal Results

A total of 100 participants who met the inclusion criteria were enrolled, and data from all participants were considered, as they fell within the predetermined target readings of HR and RR values when compared with the respective investigational devices.

### Heart Rate

**Figure 1:**
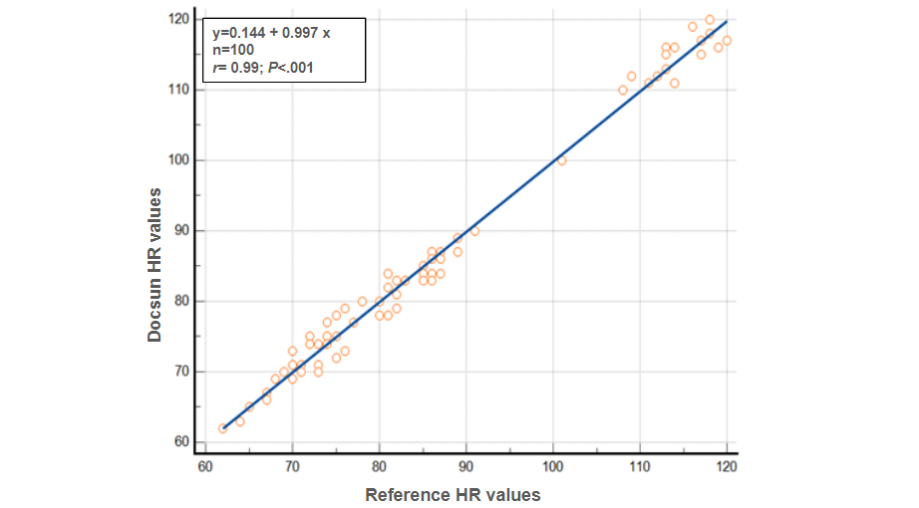
Correlation of the Docsun Telehealth Portal’s heart rate values against the reference heart rate values from a pulse oximeter.

If the HR was within ±10% of the reference standard or 5 bpm, it was considered to be acceptable for clinical purposes. The average absolute difference between the Docsun Telehealth Portal and the reference values was 1.41 (SD 1.14) bpm. The mean absolute percentage difference was 1.69% (SD 1.37). The Docsun Telehealth Portal, therefore, met the predefined accuracy cutoff for HR measurements. Correlation analysis revealed a statistically significant, strong correlation (*r*=0.99; *P*<.001) between the Docsun Telehealth Portal and the reference device values (Figure 1).

**Figure 2:**
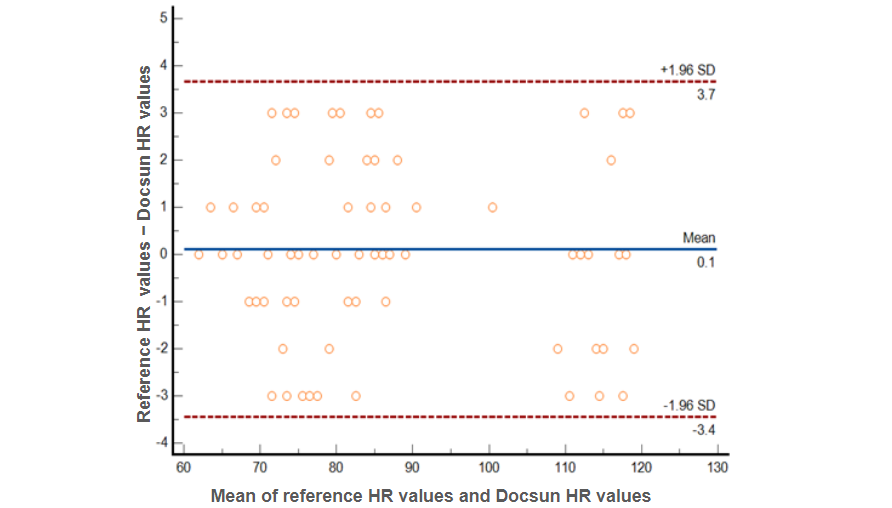
Bland-Altman plots for heart rate (HR); the reference HR was obtained from a pulse oximeter. Dots represent individual participants; blue lines indicate the mean difference; red lines indicate the 95% limits of agreement (mean 0.1, 1.96 SD).

Figure 2 shows the Bland-Altman plots comparing the HR measurements raised by the Docsun Telehealth Portal and the reference HR values raised by a pulse oximeter. All observations were within ±5 bpm. Compared to the reference HR, the mean absolute percentage error of the overall study population was 1.69%, which was significantly lower than the prespecified study target of 10%.

### Respiratory Rate

The mean difference and limits of agreement derived from the reference device and the investigational device were all within the predefined acceptance range.

**Figure 3:**
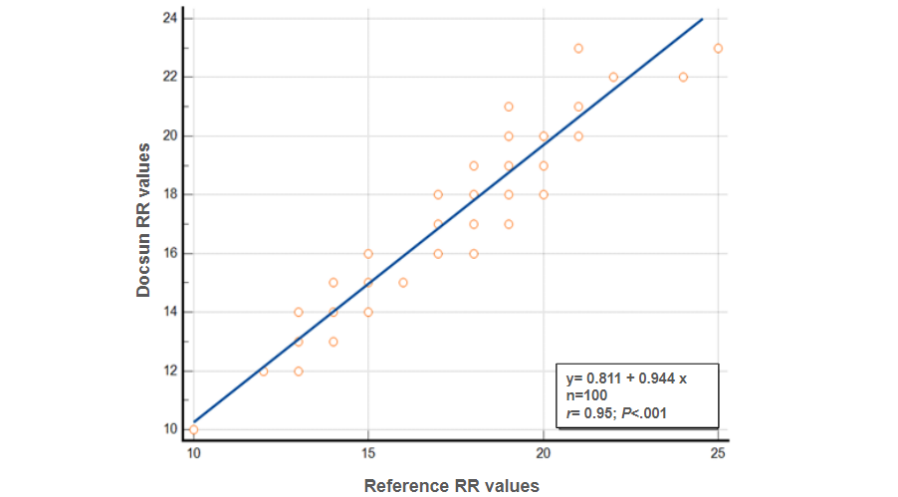
Correlation of the Docsun Telehealth Portal’s respiratory rate (RR) values against the reference respiratory rate values from a digital blood pressure monitor.

If the RR was within ±10% of the reference standard or 3 bpm, it was considered acceptable for clinical purposes. The average absolute difference between the Docsun Telehealth Portal and the reference values was 0.86 bpm. The absolute percentage difference was 4.72%. The Docsun Telehealth Portal, therefore, met the predefined accuracy cutoff for RR measurements. Correlation analysis revealed a statistically significant strong correlation (*r*=0.95; *P*<.001) between the Docsun Telehealth Portal and the reference device values (Figure 3).

**Figure 4:**
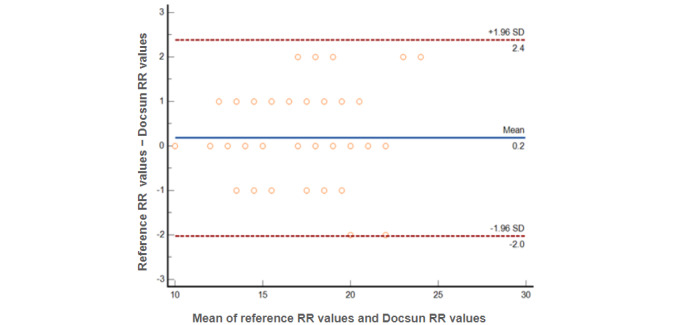
The Bland-Altman plots for comparing the respiratory rate (RR) measurements raised by the Docsun Telehealth Portal and the reference RR values raised by a digital blood pressure monitor.

Figure 4 shows the Bland-Altman plots for comparing the RR measurements raised by the Docsun Telehealth Portal and the reference RR values raised by a digital blood pressure monitor. All observations were within ±3 breaths per minute. Compared to the reference RR, the mean absolute percentage error of the overall study population was 4.72%, which was significantly lower than the prespecified study target of 10%.

### Pulse Oxygen Saturation

If the mean absolute difference between the Docsun Telehealth Portal and the reference pulse oximeter was within ±3%, it was considered accurate. The mean absolute difference in this study for SpO_2_ levels was calculated at 0.59%, which was significantly lower than the predefined study target of ±3%.

**Figure 5:**
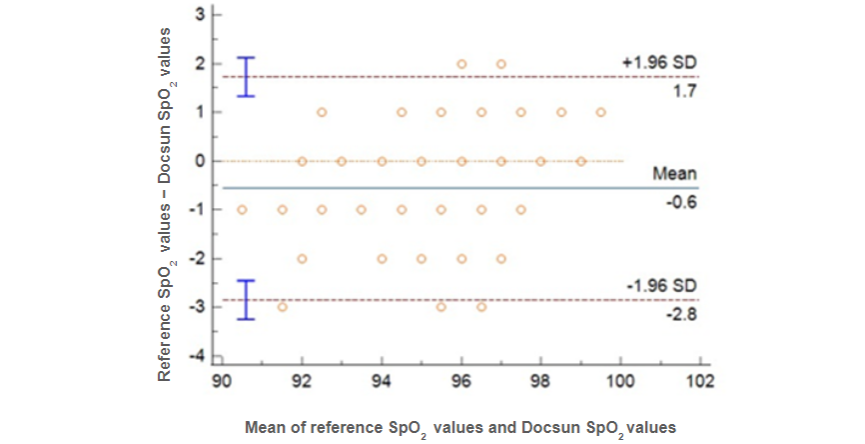
Bland-Altman plots for SpO_2_ measurements; the reference SpO_2_ values were obtained from a pulse oximeter.

**Figure 6:**
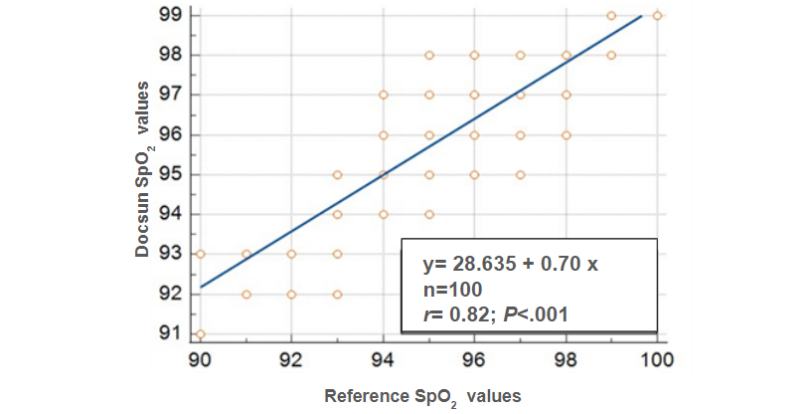
Correlation of the Docsun Telehealth Portal’s pulse oxygen saturation values against the reference pulse oxygen saturation values obtained from a pulse oximeter.

Figure 5 shows the Bland-Altman plots for comparing the reference SpO_2_ measurements with the actual SpO_2_ values. Correlation analysis revealed a statistically significant strong correlation between the Docsun Telehealth Portal and the reference values (Figure 6).

### Blood Pressure

The Docsun Telehealth Portal measured the systolic BP with an accuracy of 94.81% and the diastolic BP with an accuracy of 95.71%. The average prediction bias was 0.39 (SD 7.30) mm Hg for systolic BP and −0.20 (SD 6.00) mm Hg for diastolic BP. These SDs represent information gains of 25.5% and 12%, respectively. Our findings corresponded to average intraclass correlations of 0.60 and 0.37 for systolic BP and average Pearson correlations of 0.67 and 0.47 for diastolic BP (Table 2).

**Table 2:** The accuracy and precision of systolic and diastolic blood pressure (BP) raised by Docsun Telehealth Portal when compared with the reference BP monitor.

Model	Accuracy (%; 95 % CI)	Error bias (mm Hg; 95 % CI)	Error SD (mm Hg; 95 % CI)	Information gain vs reference SD (%; 95 % CI)	Intraclass correlation (95 % CI)	Pearson correlation (95 % CI)
Systolic BP	94.81 (94.79-94.83)	7.30 (7.28-7.32)	7.30 (7.28-7.32)	25.5 (25.3-25.7)	0.60 (0.60-0.60)	0.67 (0.67-0.67)
Diastolic BP	95.71 (95.69-95.73)	6.00 (5.98-6.02)	6.00 (5.98-6.02)	12.0 (11.7-12.3)	0.37 (0.32-0.42)	0.47 (0.46-0.48)

## Discussion

### Principal Findings

The study aimed to evaluate the accuracy of the Docsun Telehealth Portal in measuring vital signs, including HR, RR, SpO_2_, and BP, compared to reference devices. Overall, the study found that the Docsun Telehealth Portal demonstrated high accuracy in measuring these vital signs, meeting predefined accuracy cutoffs for HR, RR, SpO_2_, and BP measurements. These findings support the efficacy of the portal as a reliable tool for noninvasive vital sign monitoring, aligning with the objectives outlined in the introduction.

The detailed analysis of the study findings reveals several important insights. The accuracy of vital sign measurements provided by the Docsun Telehealth Portal has significant implications for clinical practice, particularly in remote patient monitoring and early detection of health conditions. The strong correlations observed between the portal and reference device values indicate a high level of agreement, further validating the reliability of the measurements obtained. These results are consistent with existing literature, which underscores the importance of accurate vital sign monitoring in health care delivery.

However, it is essential to acknowledge the limitations of the study. The sample size was relatively small, and data were collected from a single collaborating site, which may limit the generalizability of findings to broader populations. Additionally, while efforts were made to minimize biases and confounding factors, there may be other unaccounted variables that could have influenced study outcomes. Future research should address these limitations by conducting larger-scale studies with more diverse populations and settings.

The findings of this study have significant implications for health care delivery and patient outcomes. The accuracy and reliability of the Docsun Telehealth Portal in measuring vital signs offer a promising solution for remote patient monitoring, particularly in underserved or remote areas. By providing accessible, accurate, and timely vital sign monitoring, the portal has the potential to enhance patient care, improve health outcomes, and reduce health care disparities. Continued research and development in this area are warranted to fully realize the transformative potential of AI-based health monitoring systems in improving health care delivery worldwide.

### Conclusions

This study aimed to assess the accuracy of 4 vital sign measurements—HR, BP, SpO_2_, and RR—using the Docsun Telehealth Portal in comparison to clinically approved medical devices. The results of the study show that the accuracy guidelines for HR, BP, SpO_2_, and RR measurements were met by the investigational device when compared with the reference devices. In conclusion, the Docsun Telehealth Portal proved to efficiently and effectively provide accurate results when compared with the HR, BP, SpO_2_, and RR values measured by existing approved medical devices (ie, pulse oximeters and digital blood pressure monitors).

## Abbreviations

BP: blood pressure
Bpm: beats per minute
HR: heart rate
ICH: International Conference on Harmonization
ISO: International Organization for Standardization
RR: respiratory rate
SpO_2_: oxygen saturation

## Data Availability

The data supporting the findings of this study are not publicly available due to privacy and confidentiality reasons and applicable laws. However, data may be made available upon request from the corresponding author, subject to compliance with ethical and legal requirements.
